# The validity and reliability of the Diabetic Foot Scale-Short Form (DFS-SF) in the Turkish population: a methodological study

**DOI:** 10.55730/1300-0144.5711

**Published:** 2023-08-11

**Authors:** Meryem KILIÇ, Ayişe KARADAĞ, Neşe KOÇAKGÖL

**Affiliations:** 1 Department of Nursing, Faculty of Health Sciences, SANKO University, Gaziantep, Turkiye; 2 Department of Nursing, Faculty of Nursing, Koç University, İstanbul, Turkiye; 3 Department of Endocrinology and Metabolism, Dr. Ersin Arslan Education and Research Hospital, Gaziantep, Turkiye

**Keywords:** Diabetic foot ulcer, diabetic foot, Diabetic Foot Scale-Short Form, quality of life, validity, reliability

## Abstract

**Background/aim:**

Diabetic foot ulcers (DFUs) cause decreased quality of life due to prolonged hospital stay, loss of workforce, disabilities, psychological trauma, and increased healthcare costs. This study aims to assess the validity and reliability of the Diabetic Foot Scale-Short Form (DFS-SF) for Turkish-speaking individuals with DFUs.

**Materials and methods:**

This was a methodological study conducted with 174 Turkish patients with DFUs from March 2020 to December 2020. Translation–back translation was performed for language equivalence and expert opinions were obtained for content validity. The scale’s construct validity was tested with confirmatory factor analysis, exploratory factor analysis, and known-group validity. Cronbach’s alpha was used to evaluate internal consistency. Correlation of the DFS-SF with the SF-36 was used to test criterion validity. The scale was then revised according to the TRIPOD checklist.

**Results:**

The content validity index value was 0.93 and Cronbach’s alpha ranged from 0.93 to 0.97. The scale maintained its six-factor structure and the factor loadings ranged from 0.52 to 0.86. The fit indices of the model revealed good validity. The correlations (r = 0.43–0.76, p < 0.001) and known-group comparisons supported the construct validity.

**Conclusion:**

The Turkish version of the DFS-SF is a reliable tool for measuring the quality of life of people suffering from DFUs.

## 1. Introduction

There are half a billion people with diabetes worldwide, one-third of whom are at risk of developing diabetic foot ulcers (DFUs) in their lifetime. It is estimated that over half of all DFUs will progress to an infection, and 17% of those patients will undergo amputation [1]. Over a million people with diabetes in Türkiye suffer from DFUs, and 500,000 of them suffer from diabetic foot infections. The number of amputations caused by diabetes is approximately 12,000 annually [2].

Diabetic foot causes prolonged hospitalizations, loss of labor, disabilities, psychological trauma, and increased health care costs [3]. All of these factors reduce the quality of life of individuals with diabetes. Numerous studies have assessed the quality of life of people with foot ulcers with diabetes [4–6]. In individuals with DFUs, the quality of life is lower than that of people without diabetes [7], and it is gradually reduced in cases of nonhealing foot ulcers [8] and in individuals with DFUs. Moreover, a decrease in physical activity, lack of self-care, ulcer size, presence of infection, and polyneuropathy negatively affect quality of life [9], while religious belief positively affects it [10].

In the literature, the Short Form-36 (SF-36) is generally used to assess quality of life of people with DFUs [4–8,11–13]. However, scores obtained from the SF-36 quality of life scale may be confused with the consequences of complications other than diabetic foot. Therefore, there are doubts about its sensitivity [6]. The Diabetic Foot Scale-Short Form (DFS-SF) makes it possible to comprehensively measure the impact of DFUs on patients’ health [5,14]. This abbreviated form of the original DFS was developed to reduce patient burden and has been proven to have good psychometric properties [14]. The DFS-SF, originally produced in English, has been translated into several languages including Chinese, Dutch, French, Mandarin, Spanish, Polish, Greek, and Korean.^1^ The aim of this study is to assess the validity and reliability of the Turkish version of the DFS-SF (DFS-SF-T) to determine the quality of life of individuals with DFUs.

## 2. Materials and methods

### 2.1. Study design

The design of this study was methodological and it was conducted prospectively. The psychometric properties of the DFS-SF were tested to determine if the scale could be used in Türkiye and within Turkish-speaking communities.

### 2.2. Data collection tools

#### 2.2.1. Demographic and clinical characteristics form

A 14-item self-report questionnaire developed by the researchers was used to describe the sociodemographic (e.g., age, sex, education, type of area of residence, employment status, smoking history) and clinical (e.g., Wagner classification, wound infection, duration of diabetes, type of diabetes, hemoglobin A1c [HbA1c], previous ulcers, duration of DFU) characteristics of the individuals [2,5,15].

#### 2.2.2. Sort Form-36 (SF-36) scale

This scale was developed and put into use by Ware and Sherbourne in 1992 [15]. Its Turkish validity and reliability study was conducted by Pınar in 1995 [16]. It consists of 36 items and 8 subscales: physical function, social function, role limitations due to physical functions, role limitations due to emotional problems, mental health, energy/vitality, pain, and general perception of health. It is evaluated using a Likert-type scale (triple-six) except for items 4 and 5. Items 4 and 5 are answered as “Yes” or “No.” The subscales assess health status with scores ranging between 0 and 100, with 0 indicating poor health and 100 indicating good health [16].

#### 2.2.3. Diabetic Foot Scale-Short Form (DFS-SF)

The DFS-SF was developed to measure the impact of DFUs on patients’ quality of life. The DFS-SF is derived from the 64-item Diabetic Foot Ulcer Scale (DFS).^2^ The short form of the scale was developed by Bann et al. in 2003 [17] and contains a total of 29 items grouped into six subscales: Leisure (five items), Physical Health (five items), Dependence/Daily Life (five items), Negative Emotions (six items), Worried About Ulcers/Feet (four items), and Bothered by Ulcer Care (four items). It is a five-point Likert-type scale with answers ranging from 1 = “not at all” or “none of the time” to 5 = “a great deal” or “all of the time” or “extremely.” Domain scores are based on the sum of all items associated with that domain (raw item scores are reverse-coded when necessary). The scores per dimension are transformed on a scale from 0 to 100. A higher score indicates better quality of life [14].

### 2.3. Study process

#### 2.3.1. Language equivalence of the DFS-SF

The language adaptation of the scale was conducted following the recommendations of the World Health Organization for adapting instruments. These recommendations include forward translation, expert opinion, back translation, pilot testing, and finalization of the scale.^2^ First, the DFS-SF was translated into Turkish by two academically qualified linguists. All items were translated into a single text by the researchers and then the retranslation of the scale from Turkish to English was performed by two translators who worked independently from each other and knew both languages as native languages.

#### 2.3.2. Validity and reliability

##### 2.3.2.1. Content validity

The scale’s content validity was assessed by two academic nurses working with diabetic individuals, one diabetes nurse and one academic nurse with WOCN certification, and two academic nurses who were also diabetes nurses. Thus, a total of six experts evaluated the content validity. All expressions of the scale were rearranged in line with the recommendations of these experts. The content validity index (CVI) technique was used as a grading criterion in the evaluation of expert opinions. Each expert was asked to score the items based on a four-point Likert-type scale as follows: 4 = “very relevant,” 3 = “relevant,” 2 = “partially relevant,” and 1 = “not relevant” [20]. Furthermore, they were asked to write down any suggestions if something needed to be changed.

##### 2.3.2.2. Construct validity

Explanatory factor analysis (EFA) and confirmatory factor analysis (CFA) were used for the construct validity of the scale. For better construct validity and convergent validity, discriminant validity and known-group validity were evaluated. Significance was accepted at p < 0.05.

##### 2.3.2.3. Criterion validity

The internationally known SF-36 scale was used to determine the criterion validity. The DFS-SF-T and SF-36 scales were administered to the individuals at the same time. The correlations between the SF-36 subscales and DFS-SF-T subscales were examined. Significance was accepted at p < 0.05.

##### 2.3.2.4. Reliability

Cronbach’s alpha reliability coefficient analysis was performed to evaluate the internal consistency of the scale.

#### 2.3.1. Data collection and sample

The data were collected from patients who were taken to the diabetic foot patient clinic and hospitalized in the wound care service from March 2020 to December 2020. Patients with type 1 and type 2 diabetes who could speak Turkish and had DFUs participated in the study. In scaling sample sizes, it is recommended to reach a sample size of 5–10 times the number of items [18]. According to this guideline, 174 patients were included in the sample. Post hoc power analysis was performed after the study. In the Wagner classification system, the mean DFS-SF score and standard deviations of individuals with first- and fourth-level wounds were taken into account. When the calculation was made with effect size = 2.15, alpha = 0.05, and sample size = 174, the power was found to be 100.0%. First, a pilot study was conducted with 10 people to evaluate the comprehensibility of the scale. No changes were made to the scale items after the pilot study. Afterwards, the scale was administered to 174 patients who met the research criteria. The questionnaires were completed by the patients within approximately 15 min in face-to-face interviews.

#### 2.3.4. Data analysis

Data were analyzed using IBM SPSS Statistics 24 and SPSS AMOS-24. The data were evaluated using the Kolmogorov–Smirnov test, Q–Q graphs, and histograms. Descriptive statistical methods (mean, standard deviation, frequency, and percentage) were used to analyze the demographic and clinical characteristics of the participants. The construct validity of the scale was evaluated with CFA and EFA. Principal component analysis and varimax rotation were used in EFA. The Kaiser-Meyer-Olkin (KMO) criterion and Bartlett’s test of sphericity were used to evaluate the adequacy of the collected data. Relative goodness-of-fit indices and standardized factor loadings were evaluated in CFA. For criterion validity, the relationship between the SF-36 and DFS-SF-T was evaluated with the Spearman correlation test. For known-group validity, the differences between wound severity levels and DFS-SF-T scores were evaluated with the Kruskal-Wallis test. Cronbach’s alpha analysis was used for internal consistency analysis and the corrected item–total correlation was used for internal consistency analysis.

#### 2.3.5. Ethical considerations

Permission was received for performing the translation of the DFS-SF into Turkish. Approval was obtained from the Human Research Ethics Committee of the relevant university and from the hospital to collect the data, and written consent was obtained from the individuals who agreed to participate in the study.

## 3. Results

### 3.1. Demographic and clinical characteristics

The mean age of the patients participating in the study was 59.35 ± 8.55 years, 63.2% (n = 110) were male, 60.3% (n = 105) were primary school graduates, 68.4% (n = 119) lived in urban areas, 82.2% (n = 143) were employed, 97.1% (n = 169) had type 2 diabetes, and 52.9% (n = 92) were nonsmokers. According to the Wagner classification, 40.2% (n = 70) of the patients had grade 3 ulcers, 63.8% (n = 111) had an infection in the wound, 51.7% (n = 84) had no previous foot ulcer, the duration of diabetes was 14.72 ± 7.99 years, the HbA1c value was 10.06 ± 2.30%, and the DFU duration was 6.58 ± 12.35 months (Table 1).

### 3.2. Validity of the Turkish DFS-SF

#### 3.2.1. Content validity

In this study, the mean CVI of the scale was found to be 0.93. It is stated that CVI values should be above 0.80 [18]. Therefore, the scale shows excellent content validity.

#### 3.2.2. Construct validity

##### 3.2.2.1. Exploratory factor analysis

EFA and CFA were performed to determine the construct validity of the DFS-SF-T. The KMO value in the present study was 0.95. This value revealed that the study sample was adequate for EFA. The results for Bartlett’s test of sphericity were found to be statistically significant (χ^2^ = 7148.521, df = 231, degrees of freedom: 406, p < 0.001). This revealed that the data were suitable for EFA [19].

Varimax rotation was performed in EFA and principal component analysis for factor extraction. The cumulative explanatory power of the six factors was 87.61%. Accordingly, the explanatory power of factor 1 (WU/F, four questions) was 16.40%, the explanatory power of factor 2 (D/DL, five questions) was 16.32%, the explanatory power of factor 3 (NE, six questions) was 16.20%, the explanatory power of factor 4 (L, five questions) was 16.17%, the explanatory power of factor 5 (PH, five questions) was 14.18%, and the explanatory power of factor 6 (BUC, four questions) was 8.31% (Table 2).

##### 3.2.2.2. Confirmatory factor analysis

The model fit of the item–factor relationships obtained by EFA was tested by CFA. In CFA, many fit indices were checked to show the competence of the model tested. In this study, the normed fit index (NFI) and comparative fit index (CFI) were used to verify the relative goodness of fit, and the goodness-of-fit index (GFI) and the root-mean-square error of approximation (RMSEA) were used to verify the absolute goodness of fit. The fit index results were χ^2^ = 770.922, p < 0.001, χ^2^/df = 2.141, GFI = 0.76, root mean square residual (RMR) = 0.00, NFI = 0.90, CFI = 0.94, and RMSEA = 0.08 (Figure). The fit indices of the model confirmed good validity [20].

In CFA, convergent and discriminant validity are evaluated to determine the extent to which measures of a latent variable share their variance and how they are different from others. Accordingly, composite reliability (CR) and average variance extracted (AVE) were used in this study to evaluate the convergent validity of the scale. CR measurements are more reliable than Cronbach’s alpha, and values above 0.7 are considered reliable [21]. The CR value for each subscale of the DF-SF-T was as follows: “Bothered by Ulcer Care,” 0.94; “Dependence/Daily Life,” 0.97; “Leisure,” 0.96; “Negative Emotions,” 0.96; “Physical Health,” 0.96; and “Worried About Ulcer/Feet,” 0.96. The CR values of all subscales were thus found to be >0.7, indicating convergent validity (Table 3).

AVE evaluates the variance captured by the structure due to measurement error. A value above 0.5 is considered very good [21]. The AVE values of the subscales were as follows: “Leisure,” 0.85; “Physical Health,” 0.74; “Dependence/Daily Life,” 0.88; “Negative Emotions,” 0.83; “Worry About Ulcers/Feet,” 0.89; and “Bothered by Ulcer Care,” 0.81 (Table 3).

Discriminant validity is the extent to which a construct is truly distinct from other constructs by empirical standards. In this study, the Fornell–Larcker criterion was evaluated for discriminant validity. According to the Fornell–Larcker criterion, the square root of AVE of each construct should be higher than its highest correlation with any other construct [21]. In this study, the square root of AVE was higher than the correlations of the subscales with each other (Table 3).

#### 3.2.3. Known-group validity

This type of construct validity measures an instrument’s ability to distinguish among distinct groups [22]. For this reason, comparisons were performed for wound level (Wagner wound classification) and the subscales. As the wound level increased, the quality of life decreased significantly according to all subscales (p < 0.001) (Table 4).

#### 3.2.4. Criterion validity

The SF-36 scale was used for criterion validity. A moderate and highly significant positive correlation was obtained between the DFS-SF-T and SF-36 scales (r = 0.43–0.76, p < 0.001) (Table 5). A linear relationship existed between the two scales according to measurements that were made simultaneously.

### 3.3. Reliability of the DFS-SF-T

The internal consistency of all subscales of the DFS-SF-T was high, with Cronbach’s alpha varying between 0.93 and 0.97. The items of the subscales were not changed or deleted if the Cronbach’s alpha value of an item was 0.98. The item correlation value varied between 0.73 and 0.86 (Table 2).

## 4. Discussion

This study was performed in Türkiye to allow the use of the DFS-SF questionnaire by patients with DFU by adapting the scale to Turkish society and investigating its validity and reliability. The inclusion of disease-specific expressions in the DFS-SF reflects the fact that it is a specialized tool for individuals with DFUs.

Before using culture-specific measurement tools in a different culture, it is necessary to ensure language equivalence and to evaluate whether it is a reliable and valid tool for that society. In this study, the CVI value was calculated by submitting the scale items to expert opinions for content validity. If a scale’s CVI value is greater than ≥0.8, it indicates good content validity [23]. This can be attributed to the items of the scale being simple and understandable and the translation of the scale having been done by independent experts with expert opinions being received. Toygar et al. obtained a better content validity score (CVI: 97) [24]. In this study, although opinions were obtained from people working in the field of wound care, it can be said that the diversity of expert opinions was low. However, the CVI value, calculated as 0.93, showed excellent content validity.

The literature states that EFA, which is performed to verify construct validity, is not sufficient to verify validity alone, and it is more appropriate to perform CFA [20]. Therefore, both EFA and CFA were used in this study. Similar results were obtained for the versions of the scale prepared in different languages. In this study, scale items were collected within the framework of six factors, similarly to the original scale [14]. Similar results were obtained for versions of the scale in different languages [5,25–27]. Whether there is harmony between the factors determined in EFA and the theoretically stated factors can be investigated with CFA. CFA confirms the hypothesis that each item belongs to a particular factor [18,25]. In this study, the fit indices of χ^2^, χ^2^/df, GFI, RMR, NFI, CFI, and RMSEA were examined for CFA. The results showed an acceptable fit since the CFI and NFI values were between 0.90 and 0.95, the RMSEA value was <0.08, and the GFI value was above 0.70. Since the χ^2^/sd value was below 3, perfect fit was confirmed. The RMR does not have an absolute basis for its acceptance level, but the fit is better as this value approaches zero. The goodness of fit of the RMR value was found to be excellent in the present study [20,28]. The results of the CFA modeling obtained in this study were found to be compatible with other language versions of the scale [26,27]. In the other Turkish validity and reliability study of the scale, only EFA was performed; CFA was not performed [24]. The positive CFA results obtained in the present study are one of the strengths of this work.

In our study, convergent and discriminant validity, which are subtypes of construct validity, were evaluated. These express the validity of the unifying and discriminating power of items between subscales [21]. In the present study, the measured items showed consistency in measuring structures, and the independence between the factors was preserved. The convergent and discriminant validity of each factor of the DFS-SF-T was verified (Table 3). This result was similar to the results of De Oliveira Kaizer et al. [29].

The purpose of the internal consistency method is to calculate how much each question of a test measures the same quality. Values between 0.60 and 0.79 of Cronbach’s alpha coefficient of consistency are considered very reliable and those above 0.80 are highly reliable [23]. The DFS-SF-T showed very good internal consistency for Turkish society in this study (Table 2). Similar to our study, it was seen that Cronbach’s alpha values were at acceptable levels in evaluations of other versions of the scale [5,26,30]. Unlike this study, in the study of Raju et al., Cronbach’s alpha domain values were found to be high (0.99) [31]. Ma et al. found that the item correlation was higher in their study than in this study [27]. In another Turkish adaptation study of the scale, it was determined that the six-factor structure of the scale explained 77.09% of the scale [24]. In this study, it was determined that the item correlation, Cronbach’s alpha values, and the six-factor structure of the scale (87.61%) had better numerical values.

In the literature, different general quality-of-life scales are used for quality-of-life assessments [4–8,11–13]. One of the aims of developing the present scale was to develop a specific quality-of-life scale for patients with DFUs [14]. For this reason, the scale can be used instead of other quality-of-life scales, and it is important to test its distinguishing feature. Therefore, convergent validity, discriminant validity, and known-group validity analyses were performed in this study. Moreover, the internationally known SF-36 scale was used to determine the criterion validity. In contrast to this study, in the other study in which the validity and reliability of the DFS-SF scale in Turkish were evaluated, these analyses were not performed [24].

In this study, known-group validity was used to determine the discriminating power of the scale among different groups. The relationship between the Wagner wound level classification and the DFS-SF-T subscales was examined for the known-group validity assessment. As the wound level worsened, quality of life decreased significantly (p < 0.001) (Table 4). This result shows the sensitivity of DFS-SF in determining quality of life in general and according to changes in the ulcers. Similar results were obtained for the Japanese version of the scale [5]. In the Greek and Korean versions, wound healing and healing conditions were compared, and the findings support our study’s results [25,26].

Evaluating item–scale correlation is a way to determine the relationship between an item value and a scale value. If the item–scale correlation value is below 0.30, the item should be removed [23]. In our study, no items were removed because the item–scale correlation values were at acceptable levels. We can say that all items of the DFS-SF-T scale were found to be reliable in this analysis (Table 2). Other versions of the scale had lower or borderline correlation values compared to the current study [5,26,30,31]. Concurrent validity entails the evaluation of correlations between scores obtained by participants from the scale to be developed and their scores measured by another test that evaluates the same behavior. These correlations are expected to be positive and strong [32]. In this study, the correlation between the subscales of the DFS-SF-T and the SF-36 was positive, moderate, and at a high level, and the correlation between the DFS-SF scale and the SF-36 scale was found to be strong (Table 5). The correlation between the DFS-SF-T and SF-36 was higher in the present study than those reported for the Korean, Spanish, and Chinese versions of the scale [5,26,27,30,33]. The correlation boundaries were found to be similar to those of the Greek version [25]. Unlike this study, Raju et al. [31] found that the Malayalam DFS-SF survey areas correlated with the EQ-5D and VAS scales. The correlation values of this study are higher than those of the SF-36.

## 5. Conclusion

It is important to evaluate the quality of life of individuals with DFUs to guide the care and treatment process. The results of this study show that the original form of the DFS-SF, which has 29 items and 6 subscales, has acceptable psychometric properties. For this reason, it is suitable for use in Turkish society. According to these results, the scale can be used to evaluate the quality of life of Turkish-speaking individuals with DFUs. This study has contributed not only to the creation of a common language but also to the pursuit of more comparable studies around the world. The DFS-SF is a scale that can be easily used by healthcare professionals working with patients with DFUs and in different healthcare settings. The strengths of this study are that the DFS-SF-T can be used instead of the SF-36 quality-of-life scale and its ability to distinguish between different groups has been tested. In addition, within the framework of the results obtained in this study, it can be said that the scale has been shown to have better psychometric properties than those reported in a previous Turkish validity and reliability study. At the same time, the study had some limitations. Conducting the study during the COVID-19 pandemic prevented more patients from being recruited in terms of sample size. However, the result of the post hoc power analysis was found to be 100%. This shows the adequacy of the sample size. The sample consisted of only patients admitted to the hospital; this is another limitation of this research.

## Figures and Tables

**Figure:**
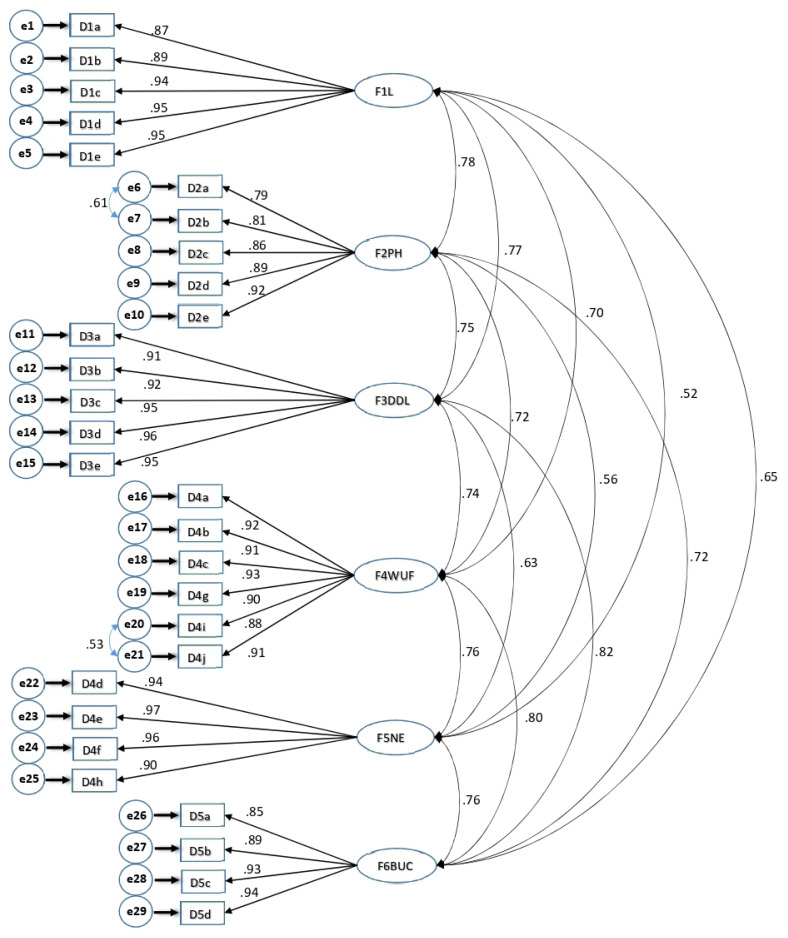
Confirmatory factor analysis. F6BUC: Bothered by Ulcer Care; F3D/DL: Dependence/Daily Life; F1L: Leisure; F5NE: Negative Emotions; F2PH: Physical Health; F4WU/F: Worried About Ulcer/Feet.

**Table 1:** Demographic and clinical characteristics of the participants (n = 174).

Demographic and clinical characteristics of the participants		n	(%)
Sex	Male	110	63.2
	Female	64	36.8
Education	Only literate	59	36.6
	Primary school	105	60.3
	High school	8	4.6
Type of residential area	Urban	119	68.4
	Rural	55	31.6
Employment	Yes	143	82.2
	No	31	17.8
Type of diabetes	Type 1	5	2.9
	Type 2	169	97.1
Smoking history	Current smoker	38	21.8
	Nonsmoker	92	52.9
	History of smoking	44	25.3
Wagner classification	Grade 1	22	12.7
	Grade 2	41	23.6
	Grade 3	70	40.2
	Grade 4	41	23.6
Wound infection	Yes	111	63.8
	No	63	36.2
Previous ulcer	Yes	84	48.3
	No	90	51.7
Age, years (range)		59.35 ± 8.55 (33–80)	
Duration of diabetes, years (range)		14.72 ± 7.99 (1–35)	
HbA1c, % (range)		10.06 ± 2.30 (4.85–15.50)	
Duration of diabetic foot ulcer, months (range)		6.58 ± 12.35 (new diagnosis–84)	

**Table 2:** Factor loading from EFA and reliability of the DFS-SF-T.

Item		F1	F2	F3	F4	F5	F6	Item correlation	Cronbach’s alpha if item removed	Cronbach ’s alpha for domain
D1A	L				0.64			0.81	0.98	0.96
D1B					0.68			0.81	0.98	
D1C					0.83			0.74	0.98	
D1D					0.85			0.73	0.98	
D1E					0.79			0.79	0.98	
D2A	PH					0.69		0.76	0.98	0.93
D2B						0.73		0.75	0.98	
D3C						0.71		0.73	0.98	
D3D						0.72		0.73	0.98	
D3E						0.73		0.76	0.98	
D4A	D/DL		0.72					0.82	0.98	0.97
D4B			0.74					0.84	0.98	
D4C			0.71					0.86	0.98	
D4D			0.74					0.84	0.98	
D4E			0.72					0.85	0.98	
D5A	NE			0.77				0.79	0.98	0.96
D5B				0.76				0.78	0.98	
D5C				0.76				0.81	0.98	
D5G				0.63				0.84	0.98	
D5İ				0.64				0.81	0.98	
D5J				0.69				0.84	0.98	
D5D	WU/F	0.79						0.75	0.98	0.96
D5E		0.86						0.73	0.98	
D5F		0.86						0.73	0.98	
D5H		0.81						0.75	0.98	0.94
D6A	BUC						0.52	0.79	0.98	
D6B							0.56	0.82	0.98	
D6C							0.68	0.81	0.98	
D6D							0.67	0.81	0.98	
Variance (%)		16.40	16.32	16.20	16.17	14.18	8.31			
Cumulative (%)		16.40	32.72	48.93	65.11	79.29	87.61			

L: Leisure; PH: Physical Health; D/DL: Dependence/Daily Life; NE: Negative Emotions; WU/F: Worried About Ulcer/Feet; BUC: Bothered by Ulcer Care.

**Table 3:** Convergent and discriminant validity of the DFS-SF-T (Fornell–Larcker criterion).

DFS-SF-T									
Measures		CR	AVE	L	PH	D/DL	NE	WU/F	BUC
DFS-SF-T	L	0.96	0.85	**0.92** a					
	PH	0.96	0.74	0.76	**0.86** a				
	D/DL	0.97	0.88	0.77	0.74	**0.94** a			
	NE	0.89	0.69	0.70	0.73	0.96	**0.91** a		
	WU/F	0.83	0.53	0.55	0.62	0.76	0.96	**0.94** a	
	BUC	0.81	0.66	0.69	0.80	0.77	0.75	0.94	**0.90** a

L: Leisure; PH: Physical Health; D/DL: Dependence/Daily Life; NE: Negative Emotions; WU/F: Worried About Ulcer/Feet; BUC: Bothered by Ulcer Care; AVE: average variance extracted; CR: composite reliability.

a Square root of AVE.

**Table 4:** Comparison of DFS-SF-T and Wagner classification.

	Grade 1 (n = 21)	Grade 2 (n = 41)	Grade 3 (n = 70)	Grade 4 (n = 41)	Test statistics^a^
	Mean ± SD	Mean ± SD	Mean ± SD	Mean ± SD	Mean ± SD
L	79.04 ± 16.48	44.26 ± 24.07	38.78 ± 24.47	22.08 ± 22.16	χ^2^ = 38.562, p = 0.000
PH	67.14 ± 26.29	43.90 ± 24.22	36.78 ± 25.10	25.73 ± 25.48	χ^2^ = 21.370, p = 0.000
D/DL	82.38 ± 20.03	47.31 ± 29.45	36.14 ± 28.43	19.87 ± 26.01	χ^2^ = 35.018, p = 0.000
NE	69.84 ± 23.23	41.66 ± 23.58	36.84 ± 22.28	26.52 ± 28.10	χ^2^ = 27.457, p = 0.000
WU/F	66.96 ± 24.53	47.71 ± 31.42	33.12 ± 27.24	22.71 ± 30.38	χ^2^ = 21.744, p = 0.000
BUC	72.91 ± 20.76	44.81 ± 26.58	30.80 ± 21.50	23.78 ± 29.38	χ^2^ = 38.581, p = 0.000

L: Leisure; PH: Physical Health; D/DL: Dependence/Daily Life; NE: Negative Emotions; WU/F: Worried About Ulcer/Feet; BUC: Bothered by Ulcer Care.

a Kruskal-Wallis test.

**Table 5:** Correlations of the DFS-SF-T and SF-36.

		DFS-SF-T						SF-36							
Measures		L	PH	D/DL	NE	WU/F	BUC	PF	RP	BP	GH	VT	SF	RE	MH
DFS-SF-T	L	1.00						0.57**	0.52**	0.62**	0.56**	0.49**	0.64**	0.48**	0.48**
	PH	0.76**	1.00					0.62**	0.51**	0.63**	0.65**	0.57**	0.61**	0.43**	0.56**
	D/DL	0.77**	0.74**	1.00				**0.76** **	0.54**	**0.65** **	0.66**	0.57**	0.73**	0.50**	0.60**
	NE	0.69**	0.70**	0.73**	1.00			0.61**	0.51**	0.64**	0.67**	**0.70** **	0.65**	0.44**	**0.72** **
	WU/F	0.53**	0.55**	0.62**	0.76**	1.00		0.75**	0.56**	0.65**	0.69**	0.64**	0.74**	**0.54** **	0.66**
	BUC	0.66**	0.69**	0.80**	0.77**	0.75**	1.00	0.70**	**0.61** **	0.62**	**0.75** **	0.68**	**0.76** **	0.50**	0.69**

L: Leisure; PH: Physical Health; D/DL: Dependence/Daily Life; NE: Negative Emotions; WU/F: Worried About Ulcer/Feet; BUC: Bothered by Ulcer Care; PF: Physical Functioning; RP: Role Physical; BP: Bodily Pain; GH: General Health; VT: Vitality; SF: Social Functioning; RE: Role Emotional; MH: Mental Health.

** p < 0.001.

## Footnotes

1 Bann CM, Fehnel SE, Gagnon DD (2003). Diabetic Foot Ulcer Scale-Short Form [online]. Website https://eprovide.mapi-trust.org/instruments/diabetic-foot-ulcer-scale-short-form. [accessed 15 December 2019].

2 World Health Organization (2008). Process of translation and adaptation of instruments [online]. Website https://www.who.int/substance_abuse/research_tools/translation/en/ [accessed 12 April 2021].
